# Supplementary material for: Evolution, systematics and historical biogeography of sand flies of the subgenus Paraphlebotomus (Diptera, Psychodidae, Phlebotomus) inferred using restriction-site associated DNA markers
Source: PLoS Negl Trop Dis. 2021 Jul 19;15(7):e0009479. doi: 10.1371/journal.pntd.0009479 (PMC8425549; doi:10.1371/journal.pntd.0009479)
Supplement: S1 Text — Matrix of geographic ranges and map of geographic regions (DOCX) [file pntd.0009479.s001.docx]

**S1 Text. Input data and results of the ancestral range estimation using BioGeoBears. Matrix of geographic ranges and map of geographic regions**

| 10 6 (A B C D E F)  *Ph_sergenti* 111111  *Ph_kazeruni* 110001  *Ph_similis* 000011  *Ph_jacusieli* 000011  *Ph_caucasicus* 000001  *Ph_mongolensis* 000001  *Ph_mireillae* 000100  *Ph_saevus* 011100  *Ph_chabaudi* 100000  *Ph_riouxi* 100000 | **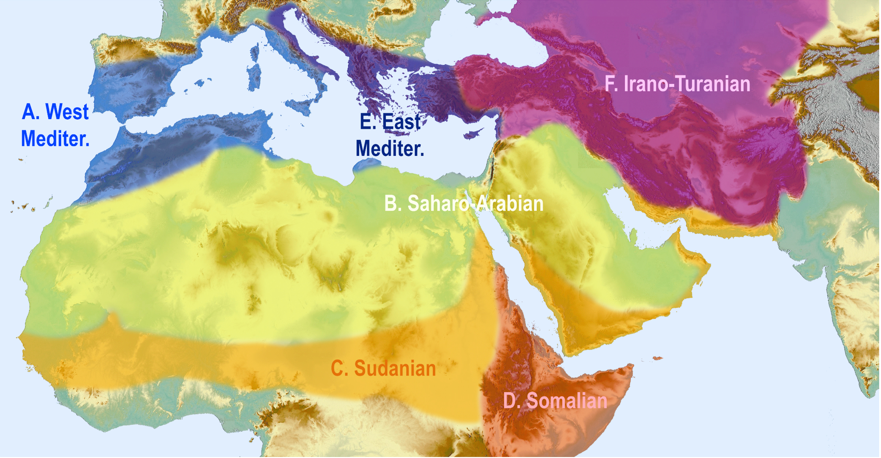** |
| --- | --- |
