# Supplementary material for: Evolution, systematics and historical biogeography of sand flies of the subgenus Paraphlebotomus (Diptera, Psychodidae, Phlebotomus) inferred using restriction-site associated DNA markers
Source: PLoS Negl Trop Dis. 2021 Jul 19;15(7):e0009479. doi: 10.1371/journal.pntd.0009479 (PMC8425549; doi:10.1371/journal.pntd.0009479)
Supplement: S2 Text — Dispersal multipliers matrices and Time Periods (DOCX) [file pntd.0009479.s002.docx]

**S2 Text. Input data and results of the ancestral range estimation using BioGeoBears. Dispersal multipliers matrices and Time Periods**

➽ : Adjacent subregions, no barrier dr=1, **X** : small water barrier dr=0.5, **X** : large water barrier or desert dr=0.1, **X** : small terrestrial area dr=0.5, **X** : large terrestrial areas dr=0.25. Probabilities are multiplied when multiple barriers occur. **A** : West Mediterranean, **B** : Saharo-Arabian, **C** : Sudanian, **D** : Somalian, **E** : East Mediterranean, **F** : Irano-Turanian.

TS2

|  |  | **A** | **B** | **C** | **D** | **E** | **F** |
| --- | --- | --- | --- | --- | --- | --- | --- |
| from -5.3 Ma  to present | **A** | - | 1 | 0.25 | 0.25 | 1 | 0.25 |
|  | **B** | ➽ | - | 1 | 0.5 | 1 | 1 |
|  | **C** | **X** | ➽ | - | 1 | 0.5 | 1 |
|  | **D** | **X** | **X** | ➽ | - | 0.25 | 0.5 |
|  | **E** | ➽ | ➽ | **X** | **X** | - | 1 |
|  | **F** | **X** | ➽ | ➽ | **X** | ➽ | - |

TS1

|  |  | **A** | **B** | **C** | **D** | **E** | **F** |
| --- | --- | --- | --- | --- | --- | --- | --- |
| from -15 Ma  to -5.3 Ma | **A** | - | 1 | 1 | 0.5 | 1 | 0.25 |
|  | **B** | ➽ | - | 1 | 0.5 | 0.1 | 1 |
|  | **C** | ➽ | ➽ | - | 1 | 0.25 | 1 |
|  | **D** | **X** | **X** | ➽ | - | 0.25 | 1 |
|  | **E** | ➽ | **X** | **XX** | **XX** | - | 0.5 |
|  | **F** | **X** | ➽ | ➽ | ➽ | **X** | - |
