# Supplementary material for: Evolution, systematics and historical biogeography of sand flies of the subgenus Paraphlebotomus (Diptera, Psychodidae, Phlebotomus) inferred using restriction-site associated DNA markers
Source: PLoS Negl Trop Dis. 2021 Jul 19;15(7):e0009479. doi: 10.1371/journal.pntd.0009479 (PMC8425549; doi:10.1371/journal.pntd.0009479)
Supplement: S3 Text — Results of model testing (DOCX) [file pntd.0009479.s003.docx]

**S3 Text. Input data and results of the ancestral range estimation using BioGeoBears. Results of model testing**

|  | **LnL** | **numparams** | **d** | **e** | **j** | **AICc** | **AICc_wt** |
| --- | --- | --- | --- | --- | --- | --- | --- |
| **DEC** | -27.07 | 2 | 0.058 | 0.0068 | 0 | 59.86 | 0.47 |
| **DIVALIKE** | -27.49 | 2 | 0.067 | 1.0e-12 | 0 | 60.69 | 0.31 |
| **BAYAREALIKE** | -29.03 | 2 | 0.047 | 0.13 | 0 | 63.77 | 0.066 |
