# Supplementary material for: Evolution, systematics and historical biogeography of sand flies of the subgenus Paraphlebotomus (Diptera, Psychodidae, Phlebotomus) inferred using restriction-site associated DNA markers
Source: PLoS Negl Trop Dis. 2021 Jul 19;15(7):e0009479. doi: 10.1371/journal.pntd.0009479 (PMC8425549; doi:10.1371/journal.pntd.0009479)
Supplement: S4 Text — Ancestral range estimations for all tested models as inferred by BioGeoBears (DOCX) [file pntd.0009479.s004.docx]

**S4 Text. Input data and results of the ancestral range estimation using BioGeoBears. Ancestral range estimations for all tested models as inferred by BioGeoBears**

**DEC.**

Left panel: Boxes at nodes show the most likely state (areas or combinations of areas).

Right panel: Pie diagrams at nodes show the relative probability of the possible states (areas or combinations of areas).

Color coding of areas is as follows:

**A** : West Mediterranean, **B** : Saharo-Arabian, **C** : Sudanian, **D** : Somalian, **E** : East Mediterranean, **F** : Irano-Turanian.

Color coding of combination of areas is determined by the program. The vertical dashed line delimits the two time periods used for the analysis.

|  |  |
| --- | --- |

**DIVALIKE.**

Left panel: Boxes at nodes show the most likely state (areas or combinations of areas).

Right panel: Pie diagrams at nodes show the relative probability of the possible states (areas or combinations of areas).

Color coding of areas is as follows:

**A** : West Mediterranean, **B** : Saharo-Arabian, **C** : Sudanian, **D** : Somalian, **E** : East Mediterranean, **F** : Irano-Turanian.

Color coding of combination of areas is determined by the program. The vertical dashed line delimits the two time periods used for the analysis.

| **** | **** |
| --- | --- |

**BAYAREALIKE.**

Left panel: Boxes at nodes show the most likely state (areas or combinations of areas).

Right panel: Pie diagrams at nodes show the relative probability of the possible states (areas or combinations of areas).

Color coding of areas is as follows:

**A** : West Mediterranean, **B** : Saharo-Arabian, **C** : Sudanian, **D** : Somalian, **E** : East Mediterranean, **F** : Irano-Turanian.

Color coding of combination of areas is determined by the program. The vertical dashed line delimits the two time periods used for the analysis.

| **** | **** |
| --- | --- |
