# Supplementary figures and images for: Evolution, systematics and historical biogeography of sand flies of the subgenus Paraphlebotomus (Diptera, Psychodidae, Phlebotomus) inferred using restriction-site associated DNA markers
Source: PLoS Negl Trop Dis. 2021 Jul 19;15(7):e0009479. doi: 10.1371/journal.pntd.0009479 (PMC8425549; doi:10.1371/journal.pntd.0009479)

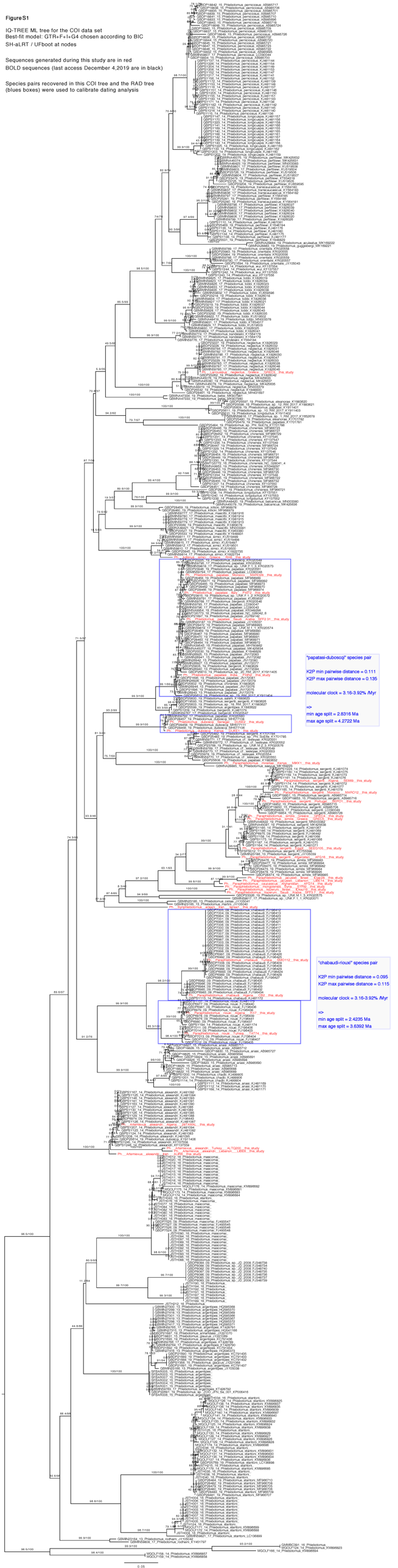

Supplement: S1 Fig — (PNG) [file pntd.0009479.s005.png]

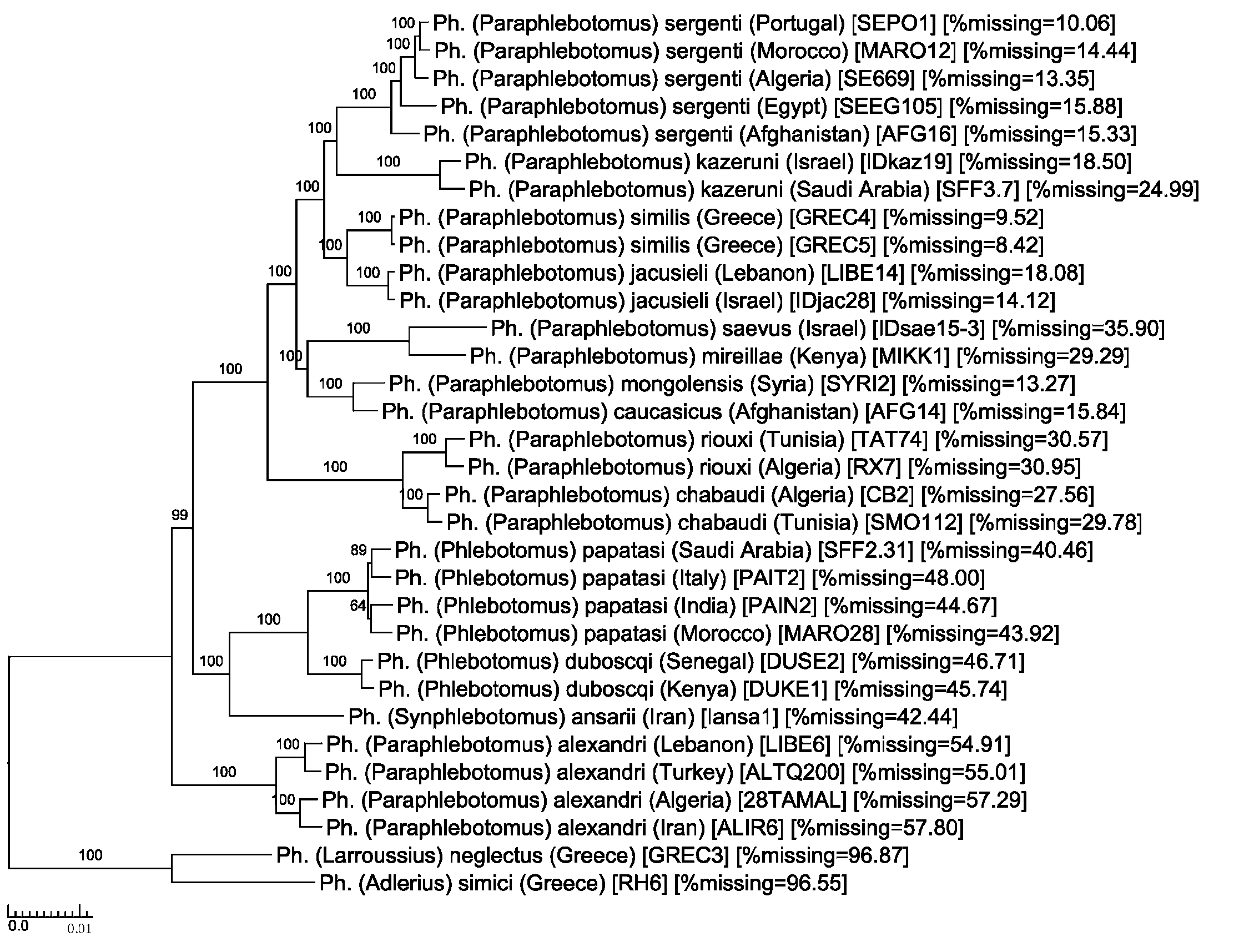

Supplement: S2 Fig — cstacks n = 6; radis_nsample_min = 50%; 5,050 loci: RAxML tree (unpartitioned data set); Bootstrap values at nodes (100 replicates); %missing = % of missing RAD tags. Data sets are described in Table 2. (BMP) [file pntd.0009479.s006.bmp]

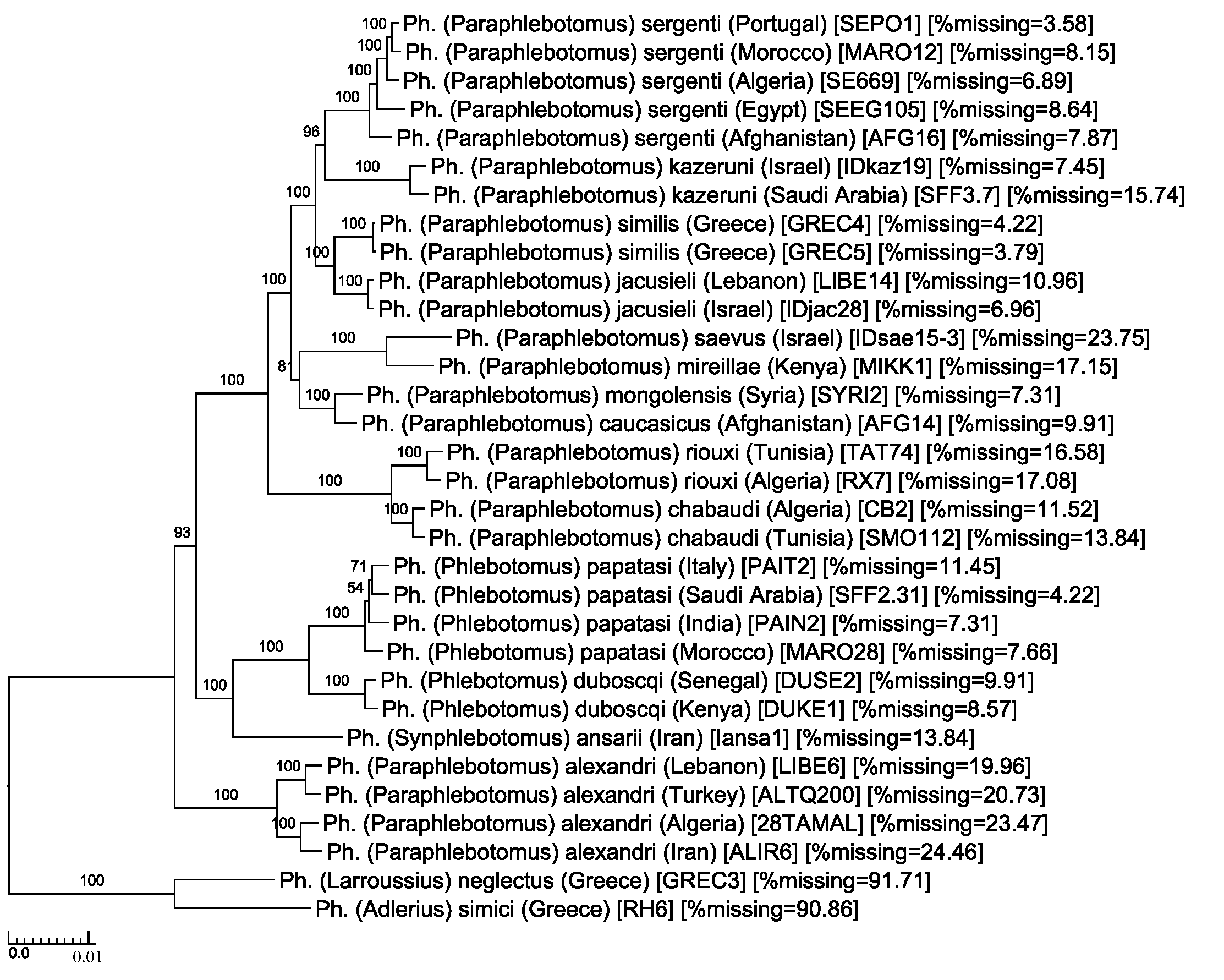

Supplement: S3 Fig — cstacks n = 6; radis_nsample_min = 75%; 1,423 loci; RAxML tree (unpartitioned data set); Bootstrap values at nodes (100 replicates); %missing = % of missing RAD tagsData sets are described in Table 2. (BMP) [file pntd.0009479.s007.bmp]

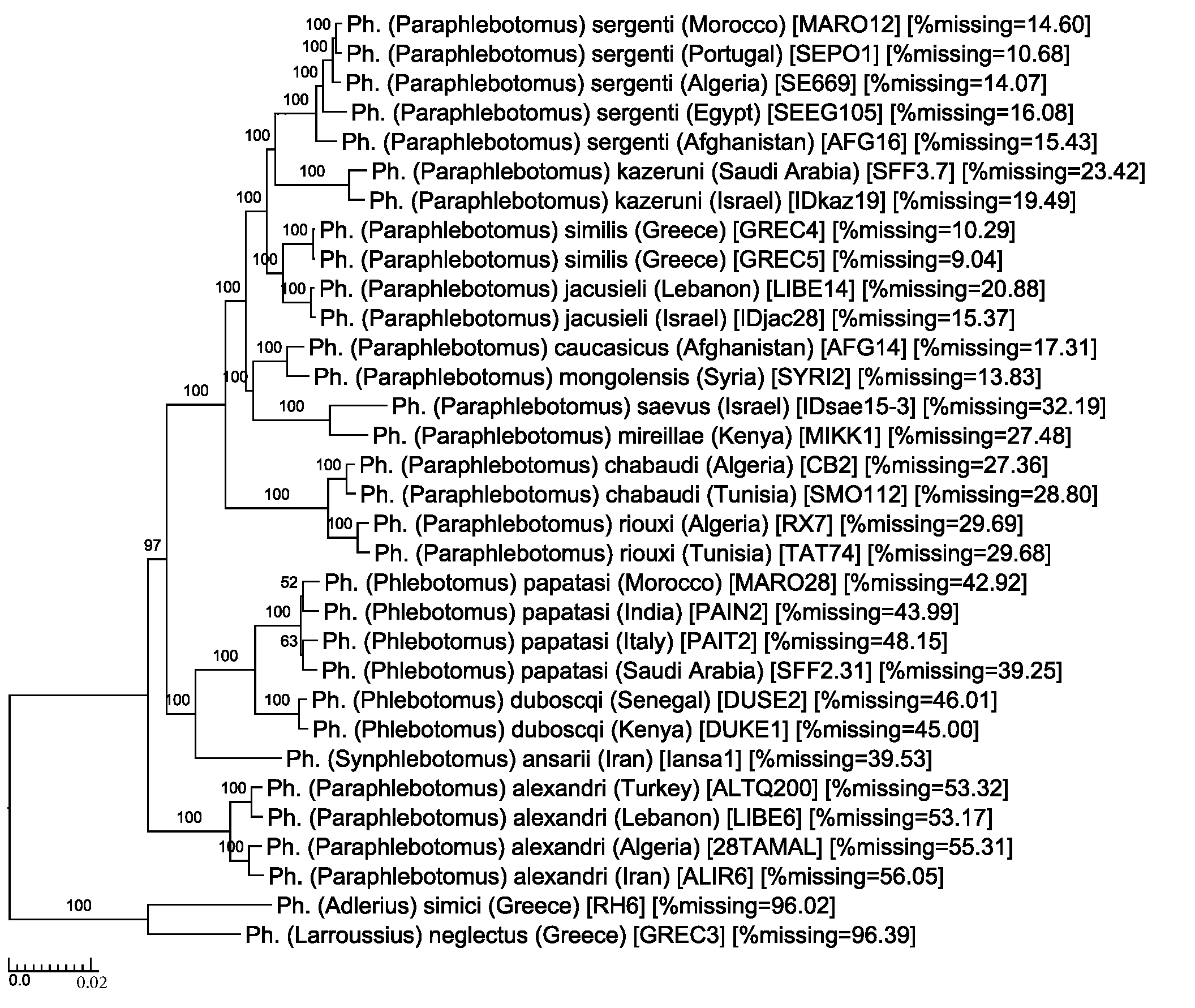

Supplement: S4 Fig — cstacks n = 8; radis_nsample_min = 50%; 7,194 loci; RAxML tree (unpartitioned data set); Bootstrap values at nodes (100 replicates); %missing = % of missing RAD tags. Data sets are described in Table 2. (BMP) [file pntd.0009479.s008.bmp]

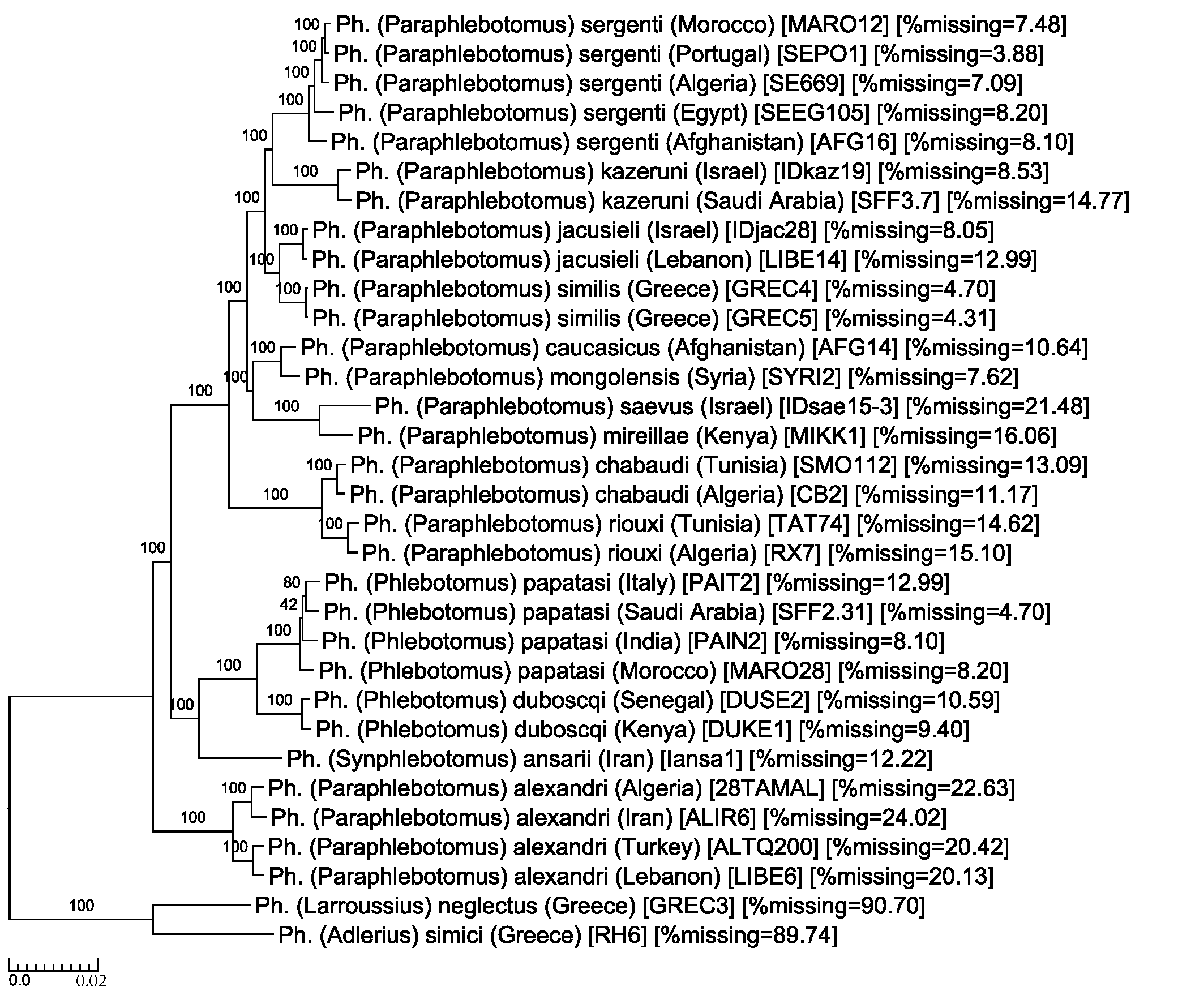

Supplement: S5 Fig — cstacks n = 8; radis_nsample_min = 75%; 2,086 loci; RAxML tree (unpartitioned data set); Bootstrap values at nodes (100 replicates); %missing = % of missing RAD tags. Data sets are described in Table 2. (BMP) [file pntd.0009479.s009.bmp]

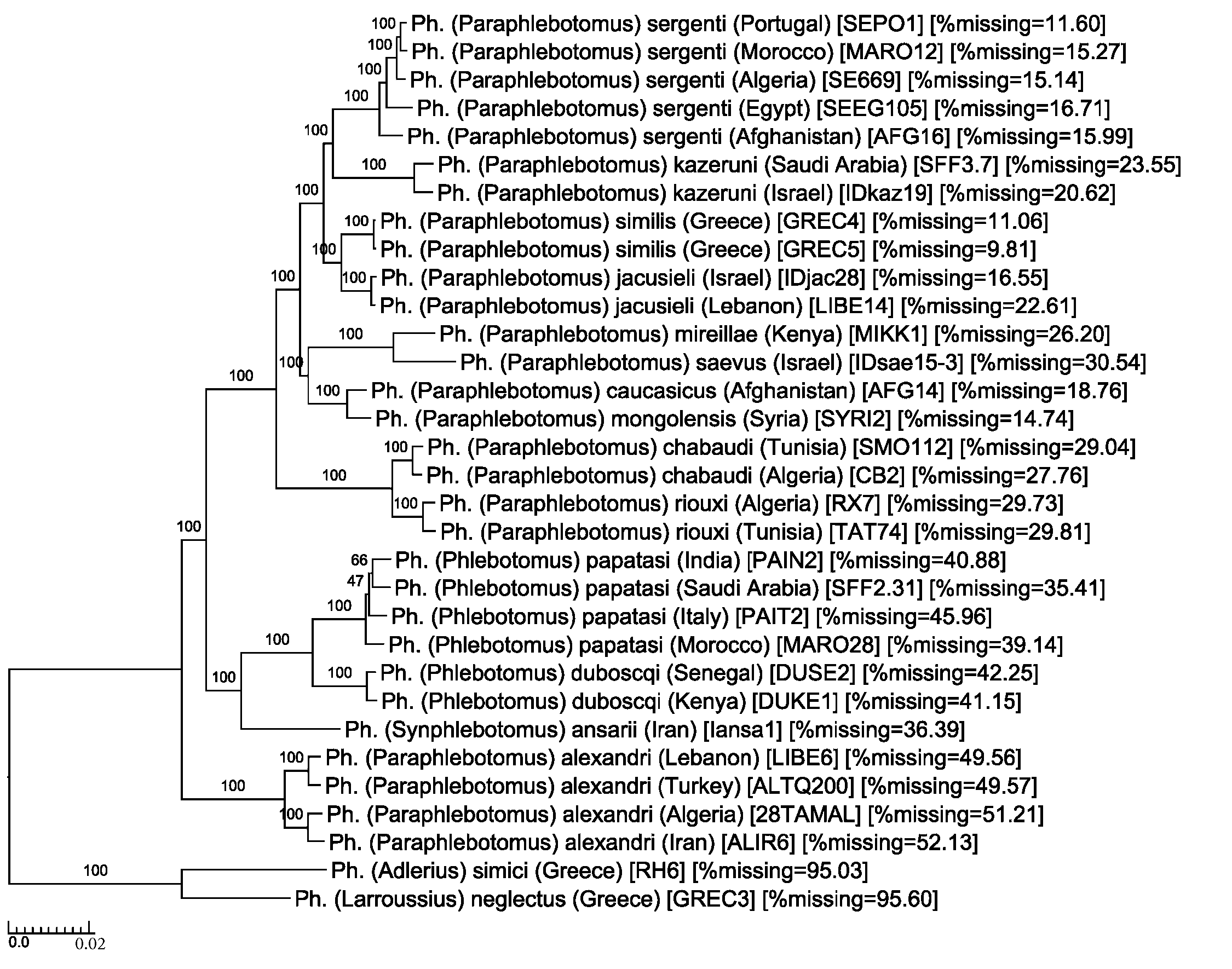

Supplement: S6 Fig — cstacks n = 10; radis_nsample_min = 50%; 8,906 loci; RAxML tree (unpartitioned data set); Bootstrap values at nodes (100 replicates); %missing = % of missing RAD tagsData sets are described in Table 2. (BMP) [file pntd.0009479.s010.bmp]

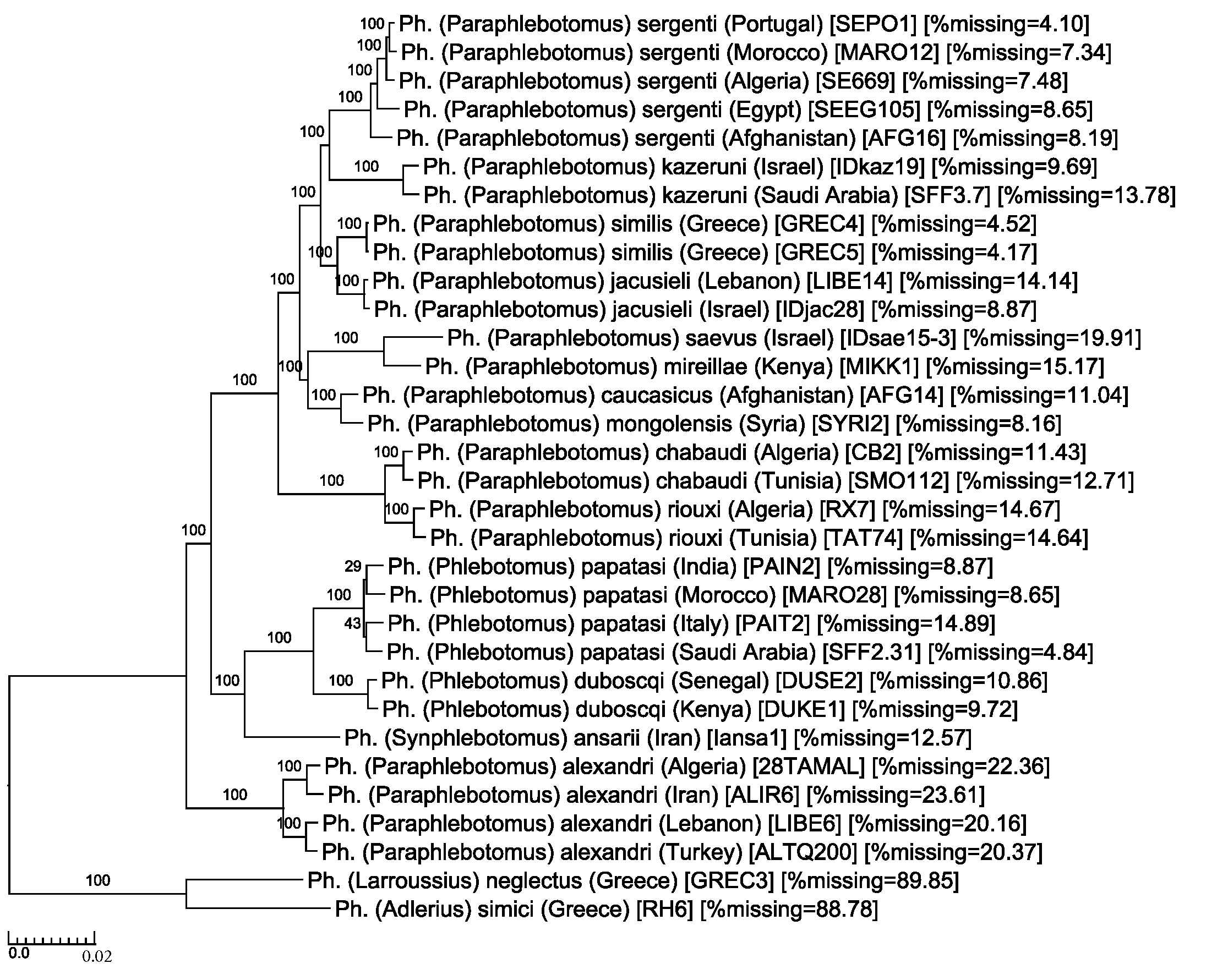

Supplement: S7 Fig — cstacks n = 10; radis_nsample_min = 75%; 2,808 loci; RAxML tree (unpartitioned data set); Bootstrap values at nodes (100 replicates); %missing = % of missing RAD tags Data sets are described in Table 2. (BMP) [file pntd.0009479.s011.bmp]
