# Supplementary material for: Evolution, systematics and historical biogeography of sand flies of the subgenus Paraphlebotomus (Diptera, Psychodidae, Phlebotomus) inferred using restriction-site associated DNA markers
Source: PLoS Negl Trop Dis. 2021 Jul 19;15(7):e0009479. doi: 10.1371/journal.pntd.0009479 (PMC8425549; doi:10.1371/journal.pntd.0009479)
Supplement: Attachment — Submitted filename: reply-to-reviewers.doc [file pntd.0009479.s013.doc]

**Jérôme DEPAQUIT**

**EA 7510 « EpidémioSurveillance et CirculAtion de Parasites dans les Environnements (ESCAPE) »**

**USC ANSES « VECPAR »**

**Laboratoire de Parasitologie**

**Faculté de Pharmacie**

**51 096 Reims cedex**

**FRANCE**

**tél. : +33 3 26 91 37 23**

[**jerome.depaquit@univ-reims.fr**](mailto:jerome.depaquit@univ-reims.fr)

**VECPAR**

Reims, April 20^th^, 2021

To Remi Charrel, associate editor

And Jaap van Hellemond, deputy editor

PloS NTD

Dear Editors,

Thank you to appreciate our paper PNTD-D-21-00283 entitled “**Evolution, systematics and historical biogeography of sand flies of the subgenus *Paraphlebotomus* (Psychodidae) inferred using restriction-site associated DNA markers.”** and to process it quickly.

Please find the requested revised version (one using the tracking change option of MS Word, and one clean version).

Please find below the relies to the reviewers comments we thank to improve the quality of our MS.

Our replies are written in red below:

**Reviewer #1**.

–line 27 Ph. alexandri, replace by Phlebotomus alexandri.

Done. Thank you.

Line 32 – give space after 5.3

Done. Thank you.

Line 142 – Nine outgroups replace with Nine outgroup specimens

Done. Thank you.

Lines 163 to 177 – replace µl with µL

Done. Thank you.

line 331 and (italic); it	shoud be non italic

Done. Thank you.

In table 1. Standardize the number of letters for the subgenera. Only Paraphlebotomus has four letters.

Because the subgenus is indicated it the table, we discarded the subgenus abbreviation.

Make table S1 available.

Done. Thank you. We forgot to download it!

As suggestion regarding the new subgenus proposed, I think it would add quality to the work if illustrations were presented on the morphological characters that make the distinction between *Paraphlebotomus* and *Artemievus* possible. I suggest illustrations of the type-species of *Paraphlebotomus* and *Phlebotomus alexandri*.

We added a new figure (figure 4) comparing *Ph. alexandri* lectotype and *Ph. sergenti* syntypes. The old figure 4 becomes the new figure 5.

Reviewer #2: The objectives are clear, the study is well design.

I just have a comment: L268-L271. I do not think this part is necessary.

We discarded a part of the sentence and maintain “Analyses were performed on the Genotoul Cluster (INRAE, France, Toulouse).”

I just have a question, L293: “16.6–34.6% missing data”. Do the authors have any idea why this rate? Is it really low 1/3 of missing data?

Yes it is, given expected loss of restriction sites with increased genetic distance (e.g. Gautier et al. 2013). I addition, with such low level it is unlikely that missing data will bias phylogenetic analyses (Wiens & Morrill, 2011)

Gautier M, Gharbi K, Cézard T, Foucaud J, Kerdelhué C, Pudlo P, Cornuet J-M, and Estoup A. 2013. The effect of RAD allele drop-out on the estimation of genetic variation within and between populations. Molecular Ecology 22:3165-3178.

Wiens JJ, and Morrill M. 2011. Missing data in phylogenetic analysis: reconciling results from simulations and empirical data. Systematic Biology 60:719-731.

Moreover, we also completed data accessibility.

Best regards,

Jerôme DEPAQUIT
